# Design and Evaluation of Synthetic Delivery Formulations for Peptide-Based Cancer Vaccines

**DOI:** 10.34133/bmef.0038

**Published:** 2024-03-21

**Authors:** Kefan Song, Suzie H. Pun

**Affiliations:** ^1^ Department of Bioengineering, University of Washington, USA.; ^2^ Molecular Engineering & Sciences Institute, University of Washington, USA.

## Abstract

With the recent advances in neoantigen identification, peptide-based cancer vaccines offer substantial potential in the field of immunotherapy. However, rapid clearance, low immunogenicity, and insufficient antigen-presenting cell (APC) uptake limit the efficacy of peptide-based cancer vaccines. This review explores the barriers hindering vaccine efficiency, highlights recent advancements in synthetic delivery systems, and features strategies for the key delivery steps of lymph node (LN) drainage, APC delivery, cross-presentation strategies, and adjuvant incorporation. This paper also discusses the design of preclinical studies evaluating vaccine efficiency, including vaccine administration routes and murine tumor models.

## Introduction

In recent decades, cancer immunotherapy has gained substantial attention as an approach that directly targets the immune system, generates less side effects, and treats cancers that are not responsive to traditional treatments. Therapeutic cancer vaccines are a type of immunotherapy that activates the adaptive immune system to fight against cancer. Sipuleucel-T, the first Food and Drug Administration-approved therapeutic cancer vaccine, treats prostate cancer by generating activated dendritic cells (DCs) displaying tumor antigen from patient peripheral blood mononuclear cells (PBMCs) and reinfusing the cells back to prime the T cells for an effective antitumor immune response [1]. However, this ex vivo process is labor-intensive, time-consuming, and cost-ineffective.

An alternative vaccination approach delivers antigens to activate antigen-presenting cells (APCs) in vivo. Several therapeutic cancer vaccines employing this strategy are currently undergoing evaluation in clinical trials [2–4]. Cancer vaccine antigens can take various forms, including tumor cell lysates [5,6], DNA [7], RNA [8], peptides [9], and proteins [10]. With defined structures and facile chemical production, peptide-based cancer vaccines offer distinct benefits compared to other forms. Peptide antigens directly serve as T cell epitopes, requiring fewer processing steps than DNA and RNA to be presented on the surface of DCs and also exhibit favorable safety profiles compared to virus-based cancer vaccines [11]. However, the relatively low immunogenicity of peptide antigens necessitates the incorporation of adjuvants to activate the adaptive immune system [12]. Additionally, peptides face challenges related to in vivo instability and susceptibility to enzyme degradation, and therefore often require carriers for delivery to desired cells.

This review addresses key considerations in the design and evaluation of peptide-based cancer vaccines, including selection of tumor antigens, development of delivery platforms, integration of adjuvants, route of administration, and preclinical evaluation models.

Tumor antigens are the key component of cancer vaccines and fall into 2 categories: tumor-associated antigens (TAAs) and tumor-specific antigens (TSAs) [13]. TAAs are antigens that are overexpressed in tumor cells [14]. Human epidermal growth factor receptor 2 is a TAA that is overexpressed in approximately 30% of human breast cancers [15]. Multiple human epidermal growth factor receptor 2-targeted peptide vaccines have shown promising results in treating patients with breast cancer [16]. Another example of a TAA is gp100, which is enriched in melanomas [17]. Patients with metastatic melanoma who received a combination of interleukin-2 and gp100 peptide cancer vaccine had improved clinical responses than patients who received interleukin-2 alone [18]. TAAs are shared among different patients, making them suitable for developing universal, cost-effective, and time-efficient treatments. However, TAAs are not uniquely expressed on tumor cells and thus are susceptible to central tolerance and dampened T cell responses [13]. In addition, cancer vaccines targeting TAAs are not completely tumor-specific and might generate toxicity in healthy tissues.

Tumor somatic mutations generate neoantigens, a subset of TSAs. Unlike TAAs, TSAs are exclusively expressed in tumor cells. They are more immunogenic and have better affinity to MHC molecules as they are not affected by central tolerance [19]. With the advancements in next-generation sequencing (NGS) and bioinformatics, neoantigens have gained increasing attention over the past years. Neoantigens can be used to create personalized cancer vaccines. Patient samples, including tumor tissue and normal tissue (PBMCs), are collected, and NGS is used to identify mutations. Algorithms and computational methods are used to predict neoepitopes for personalized vaccines [20]. Multiple peptide-based neoantigen vaccines have been tested in clinical trials [21–23]. In a phase I/Ib glioblastoma study, neoantigen vaccines containing synthetic long peptides mixed with poly-ICLC (polyinosinic and polycytidylic acid, stabilized with poly-L-lysine and carboxymethylcellulose) generated circulating neoantigen-specific CD4^+^ and CD8^+^ T cells and increased tumor-infiltrating T cells in patients not receiving dexamethasone [21]. In another phase Ib clinical trial, neoantigen peptides mixed with poly-ICLC in combination with anti-programmed death 1 (PD-1) demonstrated safety and immunogenicity in advanced melanoma, non-small cell lung cancer and bladder cancer patients [22]. Neoantigen peptide vaccines also induced long-term neoantigen-specific T cells with a memory phenotype in melanoma patients [23].

Tumor antigens are presented on tumor cell surface through major histocompatibility class I (MHC I) molecules to be recognized and eliminated by cytotoxic T cells. Many cancers have evolved to down-regulate the expression of MHC I molecules to evade T cell killing [24,25]. However, recent studies have shown that T cells are still responsible for tumor killing in antigen-negative tumor cells through Fas-dependent “bystander” killing [26] and in MHC I-negative tumors through the NKG2D–NKG2DL axis [27]. In this case, tumor killing is antigen-independent, but antigen-specific T cell activation is required, demonstrating the utility of cancer vaccines in MHC I-negative tumors.

## Mechanisms Affecting the Efficiency of Peptide-Based Cancer Vaccines

APCs are the primary cellular targets for cancer vaccines, playing a critical role in taking up, processing, and presenting tumor antigens to naïve T cells in the lymph nodes (LNs) to generate antigen-specific T cells. Activated T cells then circulate in the bloodstream to find their targets and mount an antitumoral response.

As professional APCs, DCs are target cells for antigen peptide delivery that possess a unique capability to activate naïve T cells. DCs can sense infection or endogenous danger from signals such as pathogen-associated molecular patterns and damage-associated molecular patterns. Pathogen-associated molecular patterns and damage-associated molecular patterns are detected by pattern recognition receptors expressed on DCs and lead to activation and maturation of the DCs [28]. Activated DCs up-regulate expression of costimulatory molecules on their surface and also produce proinflammatory cytokines and chemokines.

Antigens captured by the DCs are processed for presentation to T cells by 2 pathways. In the first pathway, endogenous antigens present in the cytosol are degraded into peptides by the proteasome, loaded onto major histocompatibility complex (MHC) class I molecules in the endoplasmic reticulum, and then transported to the DC surface to activate CD8^+^ T cells [29]. CD8^+^ T cells, also known as cytotoxic T cells, are directly involved in killing tumor cells. Upon antigen recognition, they release cytokines, granule-associated enzymes and death receptor-ligand engagement to eliminate cancer cells [30]. In the second pathway, exogenous antigens that are endocytosed are degraded into peptides by endosomal proteases. Vesicles containing these peptides are fused with vesicles containing MHC class II molecules, allowing peptides from exogenous antigens to be loaded onto MHC class II molecules. These loaded peptides are then transported to the DC surface to activate CD4^+^ T cells [29]. CD4^+^ T cells, also known as helper T cells, contribute to the development and maintenance of an antitumoral response [31]. In some DCs, exogenous antigens can be presented on MHC class I molecules by retrotranslocation to activate CD8^+^ T cells, a process called cross-presentation [32]. In peptide-based cancer vaccines, most peptide antigens are exogenous antigens and activate CD4^+^ T cells through the MHC class II pathway. Effective tumor cell eradication requires cross-presentation in DCs to mount strong CD8^+^ T cell responses.

T cell priming requires 3 signals [33]. First, T cell receptors (TCRs) recognize the cognate antigenic peptide presented by MHC molecules on DCs. Second, costimulatory molecules, such as B7.1 (CD80) and B7.2 (CD86) on DCs interact with CD28 on T cells [34]. Antigen recognition without costimulation can result in T cell anergy [35]. Third, cytokines released by activated DCs induce T cell expansion and differentiation [36]. Peptide antigens are known to be nonimmunogenic and are insufficient to stimulate DCs to express the costimulatory molecules required for effective T cell activation. Consequently, adjuvants, which modulate the immune response and enhance the immunogenicity of the antigen, need to be incorporated in the vaccine formulation to elicit a robust T cell response against tumor cells.

**Fig. 1.**
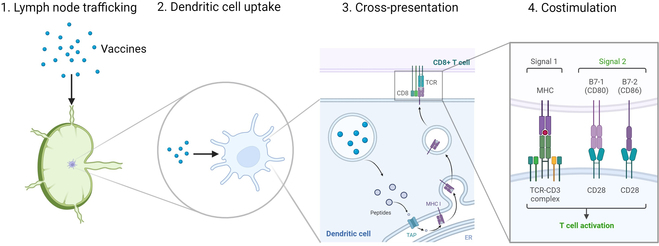
Key aspects of designing effective peptide-based cancer vaccines. Cancer vaccines need to enter the LNs, be processed by DCs, access the MHC class I pathway through cross-presentation, and induce the expression of costimulatory molecules in antigen presenting cells for effective T cell activation. Created with BioRender.com.

This review will discuss key aspects of designing effective peptide-based cancer vaccines, including LN trafficking, DC uptake, cross-presentation, and adjuvant incorporation (Fig. 1).

### LN delivery

Vaccine formulations can be delivered to peripheral, tissue resident DCs that are then activated and transmigrate into lymphatic vessels to reach LNs or drain directly into the LN for uptake by and activation of immature DCs residing in the LN [37,38]. Molecular size is one of the greatest determinants in LN delivery. When substances are administered to the interstitial space through subcutaneous, intramuscular or intradermal injection, molecules that are below 10 nm in size can access blood capillaries. Conversely, particles over 100 nm are excluded from direct diffusion into the vasculature due to the size constraints within interstitial aqueous channels [39]. Particles with hydrodynamic diameter between 10 and 100 nm are able to be transported to the LNs efficiently [40,41]. Different kinds of biomaterials including polymers [42], lipid nanoparticles [43], and inorganic nanoparticles [44] with size falling within this range have been developed for LN delivery. For instance, Lynn et al. [45] developed a peptide cancer vaccine platform with peptide-Toll-like receptor 7/8 (TLR7/8) conjugates that self-assemble into nanoparticles around 20 nm in size, irrespective of the peptide sequence. The peptide antigen was linked to a hydrophobic block on one end to facilitate particle formulation through micellization, and a charge-modifying group was incorporated on the opposite end to stabilize the nanoparticles (~20 nm in size). Synthetic long peptides in the particle form were retained longer in the draining LNs and had higher DC uptake compared to the peptides in soluble form. LN-targeting nanoparticles also elicited a stronger immune response compared to nondraining 1-μm nanoparticles or soluble antigens [46].

Particle size also affects the location of nanoparticles inside LNs and the cell types that they interact with. In one study, labeled tracers of various sizes were infused into the skin of mice. The 10- and 30-nm dextran tracers were found in the subcapsular sinus at early time points, due to direct LN drainage and size exclusion barrier of the LN conduits, whereas 500-nm particles were found deeper in the LN parenchyma at later time points as they were transported by migratory cells [47]. Particles with larger sizes (200 nm) can drain into the LN sinus, potentially due to interstitial hydrodynamic forces after injection, where they are internalized by lymphatic sinuses (LS)–DCs, which are a subset of LN-resident DCs that can generate rapid T cell responses [48]. Nanoparticles below 100 nm can get into the LN follicles, but small particles (5 to 15 nm) were cleared after 48 h, whereas large particles (50 to 100 nm) retained for over 5 weeks, generating more delivery to follicular DCs and a higher humoral immune response [49]. In addition, CD169^+^ subcapsular sinus macrophages phagocytose nanoparticles above 30 nm and suppressing those macrophages allowed more 100-nm nanoparticles to accumulate in LN follicles and enhanced the humoral immunity [50]. Tumor also affects lymphatic transport of antigens. 500-nm particles were transported to the tumor-draining LNs to a greater extent in melanoma models compared to naïve models, probably due to tumor-induced matured APCs being more migratory. In contrast, melanoma decreased 10- and 30-nm dextran tracers accumulation in tumor-draining LNs compared to drainage from normal skin [47].

Another strategy for enhancing LN delivery involves “albumin hitchhiking”. Albumin present in the interstitial fluid naturally travels to the bloodstream through the lymphatic system. Peptide-based cancer vaccines can be designed to bind to albumin and use this route to deliver antigens to the lymphatic system. Zhu et al. [51] designed an albumin-binding vaccine by conjugating thiol-modified antigen and thiol-modified CpG to maleimide-functionalized Evans Blue derivatives, which bind to human serum albumin. When administered subcutaneously, the vaccines bind to albumin in the interstitial space and are transported to the LNs. This approach also improves APC delivery as albumin can be internalized by APC through endocytosis. The results showed a 100-fold increase in LN delivery compared to the benchmark incomplete Freund’s adjuvant (IFA). Similarly, Liu et al. [52] developed amphiphiles consisting of peptide antigen and CpG DNA linked to a lipophilic albumin-binding tail. Lipid-modified CpG showed 12-fold higher LN accumulation compared to soluble CpG over 7 d after injection. Mice immunized with antigen and albumin-binding CpG adjuvant induced 32-fold higher amounts of antigen-specific T cells compared to immunization with antigen and unmodified CpG.

### DC-targeting strategies

**Fig. 2.**
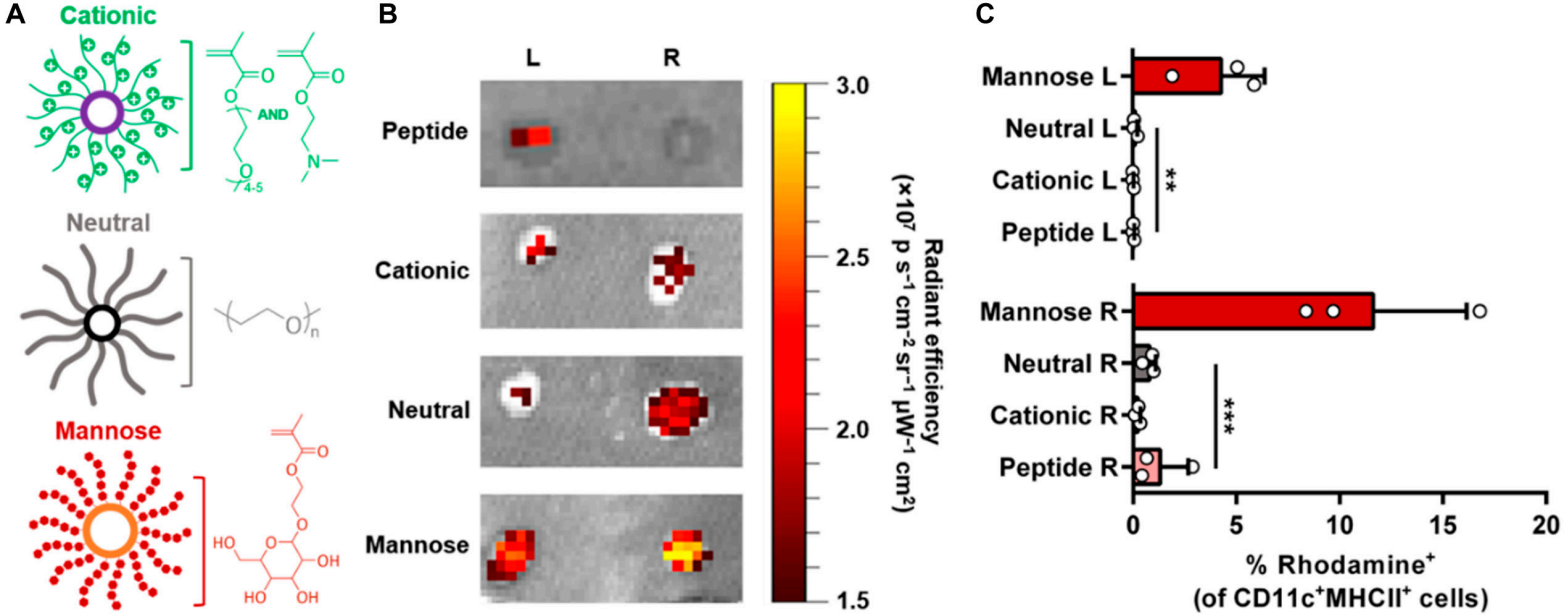
(A) Cationic, neutral and mannose micelle structures. (B) Fluorescence imaging of right and left draining inguinal LNs (48 h posttreatment). Mannosylated micelles are retained more efficiently in LNs. (C) Flow cytometry analysis of LN-resident cells confirms that mannose-micelle formulations are internalized by CD11c^+^MHCII^+^ DCs. Data are presented as mean ± SD. *N* = 3 biological replicates. Statistical significance was calculated using one-way ANOVA with post hoc Fisher’s least significant difference test (**P ≤ 0.01, ***P ≤ 0.001). Reprinted with permission [57].

Targeting ligands that bind to receptors preferentially expressed by DCs can facilitate vaccine uptake through receptor-mediated endocytosis. The mannose receptor (CD206) is a C-type lectin that is highly expressed on immature DCs [53]. Both liposomes and polymers have been functionalized with mannose to enhance peptide delivery to DCs [54–57]. For instance, Lv et al. [57] compared polymeric micelles with same micellar core but different coronas (PEGylated, cationic or mannosylated) and demonstrated higher inguinal LNs accumulation of the mannosylated micelles and 16-fold higher DC internalization than PEGylated micelles and 79-fold higher DC internalization than cationic micelles (Fig. 2) CD40 is a transmembrane receptor expressed on APCs. Rosalia et al. [58] encapsulated antigens and adjuvants in poly(lactic-co-glycolic acid) and coated the nanoparticles with an agonistic αCD40-mAb to target DCs. Similarly, Trabbic et al. [59] coated gold nanoparticles with β-1,3-glucans (B13G) to target Dectin-1 on APCs.

## Strategies to Enhance Cross-Presentation

### Utilizing pH-responsive biomaterials for endosomal membrane disruption

**Fig. 3.**
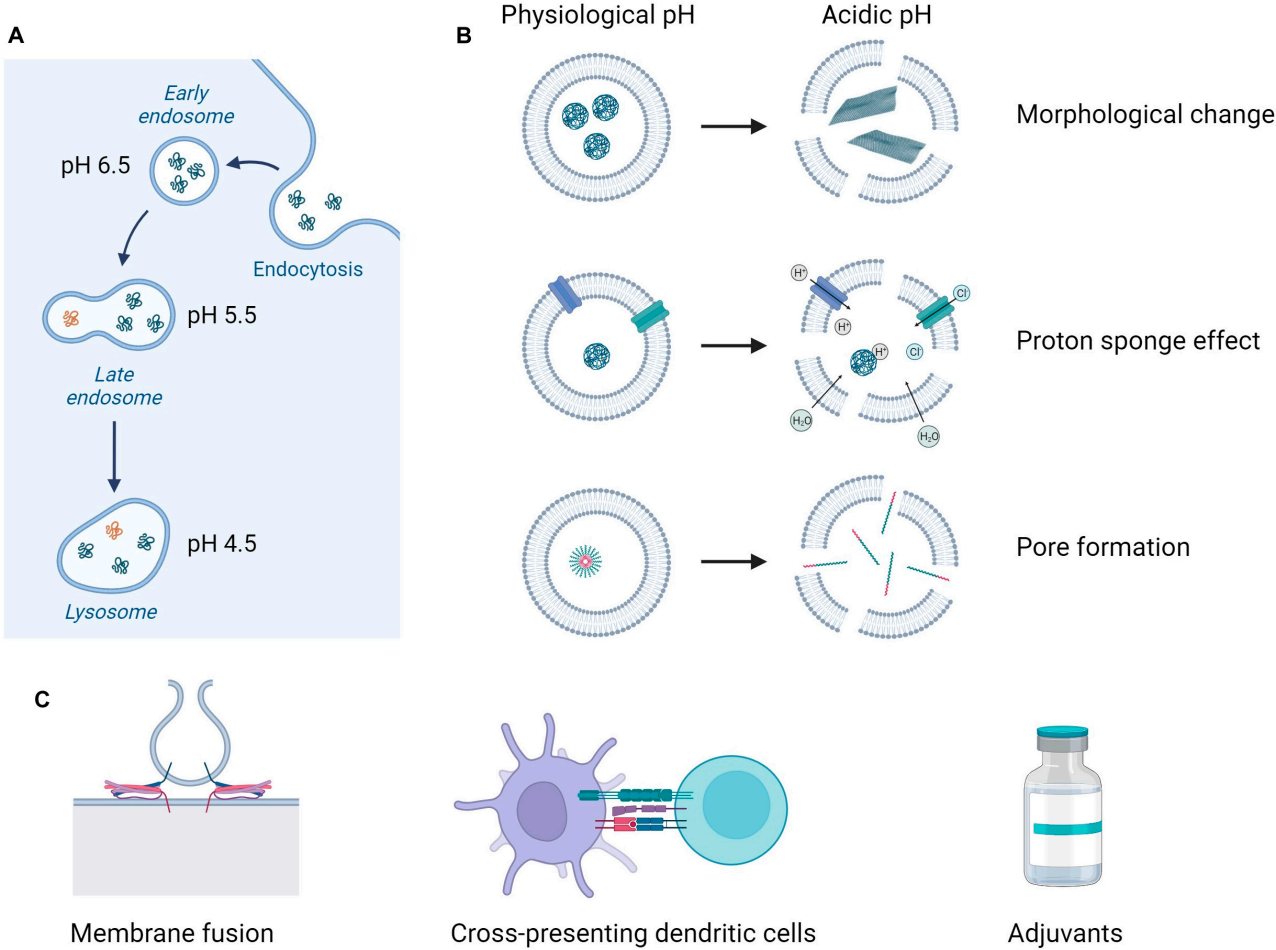
Strategies to enhance cross-presentation. (A) pH drops as endosomes mature into lysosomes. (B) pH-responsive biomaterials that utilize the acidification within the endosome-lysosome system to disrupt membranes. (C) Other methods to enhance cross-presentation, including membrane fusion for direct cytosolic delivery, targeting cross-presenting DCs, and using adjuvants with cross-presenting activity. Created with BioRender.com.

Many peptide-based vaccine formulations enter cells through endocytosis and therefore need to escape the endosome and reach the cytosolic compartment for MHC class I presentation to CD8^+^ T cells. As endosomes mature into lysosomes, pH levels drop from 6.5 in early endosomes to 4.5 in lysosomes to activate lysosomal enzymes (Fig. 3A) [60]. Various biomaterials have been designed to leverage the acidification within the endosome-lysosome system, leading to the development of pH-responsive biomaterials (Fig. 3B). Ideally vaccine formulations will selectively facilitate endosomal membrane disruption and avoid toxicity associated with cell membrane disruption.

One method to facilitate endosomal escape involves inducing morphological changes to mechanically disrupt the endosome. Gong et al. [61] developed a proton-driven nanotransformer-based vaccine, by conjugating pyrene-conjugated ᴅ-peptide (PDP) to an amphiphilic block copolymer via an acid-cleavable acetal bond. Under physiological pH conditions, these polymer-peptide conjugates self-assemble into nanospheres about 100 nm in size. As the pH drops in the endosome, the acetal bond hydrolyzes, releasing PDP molecules that assemble into nanosheets due to strong π–π interactions. The nanosheets are several micrometers in length or width and mechanically disrupt the endosomal membrane to release peptide antigens into the cytosol. Another method employs changes in hydrophobicity to promote endosomal escape. The Stayton group [62] employed endosomolytic polymers to deliver antigens, improving CD8^+^ T cell response compared to control formulations. The Wilson group [63] developed electrostatically stabilized nanoparticles by mixing decalysine-modified peptides with poly (propylacrylic acid). At acidic pH, poly (propylacrylic acid) switched from hydrophilic to hydrophobic and destabilized the endosomal membrane to promote cytosolic delivery of the peptide antigen.

Another method to disrupt the endosomal membrane is through the “proton sponge” effect. This concept posits that buffering polycations swell as they are protonated and induce more protons and chloride ions to the endosome. The resulting osmotic pressure and polymer swelling cause endosomal rupture [64], although the exact mechanism remains a subject of debate. Cationic polymers with secondary or tertiary amines are often used to create a proton sponge effect [65]. Luo et al. [42] generated a library of pH-sensitive nanoparticles containing copolymers with tertiary amines in linear or cyclic side chains and discovered that a polymer called PC7A, with a tertiary amine in a cyclic 7-membered ring exhibited a high level of cross-presentation when applied for vaccine delivery. This polymer also demonstrates adjuvant activity. Zhou et al. [55] synthesized an amphiphilic diblock copolymer, PEG-b-PDPA, with a sharp pH transition at 6.3, and combined it with a cationic polymer, OEI-C14, to facilitate endosomal escape through the proton sponge effect.

Finally, molecules like perforin can form pores in the endosomal membrane to release biomolecules into the cytosol [66]. This process naturally occurs in conventional DC1 (cDC1) for cross-presentation. Pore-forming molecules such as lytic peptides can be used to facilitate endosomal disruption. In a recent study in the Pun group [67], a bee-derived lytic peptide ᴅ-melittin was conjugated to a pH-sensitive polymer that self-assembled at physiological pH and dissociated at acidic endosomal pH. As pH drops in the endosome, the lytic peptide is exposed destabilizing the endosomal membrane, facilitate peptide antigen release, and promote cross-presentation.

### Reactive oxygen species

Reactive oxygen species (ROS) cause lipid peroxidation in DCs, which disrupts the endosomal membrane and facilitates antigen release into the cytosol for cross-presentation [68]. Xu et al. [69] developed a PEGylated reduced graphene oxide nanosheet (RGO-PEG) and conjugated neoantigen peptides through maleimide reactions and adsorbed CpG to the nanosheet via π–π stacking. RGO-PEG increased intracellular ROS levels in DCs and enhanced cross-presentation of the neoantigen peptides.

### Direct cytosolic delivery

Peptides that are delivered directly to the cytosol through membrane fusion may avoid endosomal entrapment. SNARE proteins mediate synaptic vesicles and plasma membrane fusion during exocytosis [70]. Yang et al. [71] developed complementary lipidated coiled-coil peptides and modified cell membranes and liposomes with the complementary to achieve targeted membrane fusion and intracellular delivery. However, as cell membrane fusion requires modification to the cells, further progress is needed for in vivo applications like cancer vaccines.

### Targeting cross-presenting DCs

DCs have 2 subtypes: cDCs and plasmacytoid DCs [72]. cDCs are further divided into 2 subsets: cDC1 and cDC2. cDC1 are known to be potent at cross-presentation [73]. Their high expression of MHCI, high ROS production and low pH contribute to enhanced cross-presentation [72]. In addition, a recent study showed that cDC1 expresses perforin-2, which forms pores in the endosomes to facilitate antigen delivery to the cytosol and enhance cross-presentation [74]. cDC1 express certain markers, including XCR1 (chemokine receptor), DNGR-1/CLEC9A (C-type lectin receptor), CD103 in mice (integrin αE) and BDCA3 in humans, which can be used to target cDC1 [75]. Yan et al. [76] identified a peptide, WH, that binds to Clec9a. They fused the WH peptide or a control peptide with the ovalbumin (OVA) antigenic peptide and observed enhanced activation of OVA-specific CD8^+^ T cells with the WH-fused peptide compared to the control peptide. This study demonstrated that peptide WH could bind to Clec9a and targeting cDC1 for enhanced cross-presentation. Similarly, Arabpour et al. [77] developed fusion vaccines by fusing antigens with anti-CD103 antibodies, specifically targeting CD103^+^ cDC1, and demonstrated that CD103 targeting increased the number of antigen-specific CD8^+^ T cells.

### Adjuvants to enhance cross-presentation

Certain adjuvants facilitate antigen cross-presentation. Aluminum-containing adjuvants have been used clinically for many years and demonstrate excellent safety profiles. Although traditional aluminum adjuvant is insufficient for cross-presentation, Li et al. [78] found that when antigens were conjugated to α-Al_2_O_3_ nanoparticles, cross-presentation significantly improved through the autophagy pathway. Nanoparticles such as α-Al_2_O_3_ can induce autophagy [79] and the activation of autophagy promotes DCs to cross-present antigens to CD8^+^ T cells [80]. Saponin-based adjuvants, glycosides isolated from the South American soapbark tree, also induce cross-presentation. Huis in ’t Veld et al. [81] showed that saponin-based adjuvants promote cross-presentation through the induction of lipid bodies and activation of the PKR-like endoplasmic reticulum kinase pathway. Some adjuvants targeting the TLRs and nucleotide-binding and oligomerization domain (NOD)-like receptors expressed by DCs promote cross-presentation. Zhao et al. [82] utilized *meso*-2,6-diaminopimelic acid, a NOD1 agonist, in their polymeric vaccine formulation vaccine design to promote antigen cross-presentation in DCs.

## Considerations in Designing Vaccine Adjuvants

Adjuvants have long played a critical role in enhancing the antigen-specific responses in vaccines, and are particularly important for peptide-cancer vaccines since peptide antigens are usually nonimmunogenic on their own. While some delivery platforms such as the previously described PC7A polymer inherently possess adjuvant properties [42], other platforms require the incorporation of adjuvants to augment the immune response. Commonly used cancer vaccine adjuvants are summarized in Table 1. Aluminum salts and IFA were often used in earlier vaccine formulations to generate an antigen depot at the injection site to prolong antigen presentation. However, their adjuvant effect is relatively weak, and they promote Th2 responses, which is less suitable for cancer vaccine applications [83]. TLR agonists and stimulator of interferon genes (STING) agonists are used in more recent vaccine formulations and enhance the immune responses by generating proinflammatory cytokines [84], contributing to DC maturation [85], enhancing cross-presentation [86], and improving antigen-specific CD8^+^ T cell survival [87]. However, high levels of proinflammatory cytokines in the systemic circulation raise safety concerns. To develop effective vaccine adjuvants, the following important considerations need to be taken into account to maximize adjuvant benefits while minimizing potential drawbacks: controlling the delivery of adjuvants, optimizing adjuvant codelivery with antigens, and exploring combinations of multiple adjuvants.

**Table 1.** Common cancer vaccine adjuvants, pathways, receptor locations, and applications in peptide-based cancer vaccines

Adjuvants	Pathway / mechanism of action	Receptor location	Peptide cancer vaccine applications
Aluminum salts	Depot effect [133], NLRP3 inflammasome [134]	N/A	[135]
Incomplete Freund's adjuvant (IFA) (e.g., Montanide ISA-51)	Depot effect, cytokine and chemokine induction [136]	N/A	[137]
Granulocyte-macrophage colony stimulating factor (GM-CSF)	DC maturation, T cell activation [138]	Cell surface	9
CpG oligodeoxynucleotides(CpG ODNs)	TLR 9 pathway	Endosome	[51,98]
polyinosinic acid: polycytidylic acid Poly(I:C)	TLR 3 pathway	Endosome	[97, 127]
Imidazoquinoline derivatives (IMDs)(e.g., resiquimod, imiquimod, and gardiquimod)	TLR 7/8 pathway	Endosome	[45, 128]
Stimulator of interferon genes (STING) agonists (e.g., cGAMP, ADU-S100)	STING pathway	Endoplasmic reticulum	[55, 95]

### Controlled delivery of adjuvants

Despite recent advances in adjuvant development, uncontrolled delivery of adjuvants may generate proinflammatory cytokines in the systemic circulation and cause toxicity [88]. To address this problem, researchers have developed pH-responsive and redox-responsive materials to control the release of adjuvants. Aichhorn et al. [89] developed a pH-sensitive polymeric prodrug with hydrazide linkers that releases TLR 7/8 agonist in the acidic endosomal environment. This polymer accumulated in endosomal and lysosomal vesicles and adjuvant release was greatly enhanced at pH 5 compared to pH 7.4. Similarly, Zhou et al. [55] developed a DC-targeted diblock copolymer that formed micelles at physiological pH but disassembled when pH was below 6.3. They conjugated DMXAA, a STING agonist to the polymer backbone through an esterase-labile bond so that the STING agonist is released inside DCs rather than in the blood circulation. Nguyen and Song et al. [90] reported a polymeric STING adjuvant formulation with STING agonist-3 conjugated to the polymer backbone via a cathepsin substrate which is cleaved in endosomal compartments. Bhagchandani et al. [91] expanded on this concept by creating a library of R848 bottlebrush prodrugs with different aryl ester linkers to achieve release half-life ranging from 4 to 40 d. They noticed that the fast release prodrug had similar maximum tolerable dose compared to free R848, while the medium and slow release prodrug had maximum tolerable doses 4 and 8 times higher than the free drug when administered intravenously. The medium and slow release prodrugs slowed tumor progression and improved survival compared to free R848 and displayed no signs of toxicity.

### Codelivery of antigen and adjuvant

Since antigen recognition by TCR in the absence of the costimulatory signal can induce T cell anergy [92], efficient T cell activation relies on the colocalized delivery of both antigen and adjuvant to the same DC to ensure that antigen is presented along with the necessary costimulatory molecules to activate T cells. Delivering antigens with tolerogenic immunomodulators can even induce antigen-specific immune tolerance and inhibition of T cell activation [93]. Fischer et al. [94] showed that colocalized delivery of antigen and adjuvant generated higher antibody titers compared to coadministration of antigen and adjuvant. In addition, they were able to reduce the adjuvant doses by 10-fold with colocalized delivery, potentially reducing the side effects associated with adjuvants. Antigens and adjuvants can be codelivered by coencapsulation [58,95] or by covalent conjugation on the same delivery platform [45,54].

### Combination of multiple adjuvants

Multiple adjuvants can be combined to activate different immune pathways to boost the immune response. Tom et al. [96] conjugated pyrimido-indole (TLR4 agonist), loxoribine (TLR7 agonist) and CpG-ODN (TLR9 agonist) together to form a triagonist compound. The triagonist compound generated higher immune cell activation and antibody responses compared to diagonist compounds and unconjugated mixture of the agonists, perhaps due to the proximity of agonists with covalent conjugation. Controlling the spatial arrangement of multiple adjuvants generated synergistic effects and shifted the immune response. Besides spatial control, temporal control of multiple adjuvants also plays an important role. Jin et al. [97] developed a liposome platform to codeliver poly(I:C), a TLR3 agonist, and resiquimod, a TLR7/8 agonist. Resiquimod was delivered as an inactive prodrug by conjugation to cholesterol. Poly(I:C) rapidly activated the TLR3 pathway, while TLR7/8 pathway activation was delayed for 4 to 6 h as resiquimod activity was restored by γ-interferon-inducible lysosomal thiol reductase (GILT) in the endolysosome through linker cleavage. This optimized order and kinetics of pathway activation generated non-exhausted DCs and CD8^+^ T cells and created strong antitumor immunity.

## Routes of Administration

### Subcutaneous and intramuscular administration

Subcutaneous administration is the most commonly used method for delivering peptide-based cancer vaccines in recent preclinical studies. Many studies have demonstrated that nanoparticles with diameter less than 100 nm accumulate in the LNs without any active-targeting strategies when administered through the subcutaneous route [42,69,95,97–99]. In addition, vaccines can benefit from the depot effect of this route, which prolongs the release of antigens to enhance the immune response [100]. Baharom et al. [101] developed a polymer-based nanoparticle platform delivering neoantigens and TLR7/8 agonists and found that subcutaneously administered nanoparticles can be detected at the injection site as well as LNs for up to 2 weeks, resulting in prolonged antigen presentation and a high magnitude of antigen-specific CD8^+^ T cells. However, vaccine depots can be a double-edged sword. Hailemichael et al. [102] showed that chronic antigen presentation by antigenic peptide in IFA induced T cell sequestration and deletion at the vaccination site and was less effective than a short-lived water-based formulation. While the subcutaneous route is often used for preclinical cancer vaccine studies, the intramuscular route is preferred in clinical settings as it allows for rapid absorption, large injection volume, and reduced irritation. Kuai et al. [103] evaluated a cancer nanodisc vaccine platform by both subcutaneous and intramuscular injections, and demonstrated that cancer vaccines administered through the subcutaneous route had higher LN delivery and induced more antigen-specific T cells compared to the intramuscular route. Currently, subcutaneous delivery of cancer vaccines is still the preferred route for most preclinical studies.

### Intradermal administration

Intradermal delivery is another suitable option for peptide-based cancer vaccines. The papillary dermis, where this route targets, is highly vascularized and contains many APCs such as Langerhans cells and DCs, which are good targets for cancer vaccines [104]. Kim et al. [105] developed dissolving microneedles with amphiphilic triblock copolymer, Pluronic F127, hydrophobic resiquimod adjuvant, and hydrophilic antigen. The microneedles dissolve in the intradermal fluid, forming micelles that encapsulate the antigen and adjuvant, and then travel to the LNs to activate the DCs.

### Intranodal administration

Intranodal delivery allows cancer vaccines to be delivered directly to the LNs. Senti et al. [106] showed that intranodal delivery of allergens generated less side effects compared to subcutaneous delivery in a clinical trial. Intranodal injection of peptide antigens generated a higher CD8^+^ T cell response compared to subcutaneous and intradermal vaccinations possibly due to short half-life and inefficient delivery of free peptides when administered outside of the lymphatic system [107]. It also induced a higher immunoglobulin G2a response and inteferon-γ production compared to subcutaneous, intramuscular, and intradermal administrations [108]. However, this method requires ultrasound guidance in patients [109] or requires surgically exposing the LNs in mice [110] and is more invasive than subcutaneous and intradermal injections.

### Intravenous administration

Treatments using the intravenous route offers some advantages for vaccine applications but pose higher risk of systemic toxicity. Baharom et al. [101] compared intravenous and subcutaneous routes for delivery of a nanoparticle containing neoantigen and TLR7/8 agonist. At the same antigen dosage, subcutaneous administration generated higher frequency of antigen-specific CD8^+^ T cells than intravenous administration, which could be due to prolonged antigen retention by the subcutaneous route. However, the intravenous route generated antigen-specific CD8^+^ T cells that were more stem-like, whereas the subcutaneous route generated antigen-specific CD8^+^ T cells in the effector state. When the intravenous dosage was increased by 4-fold to match the magnitude of CD8^+^ T cell response generated by the subcutaneous administration, the investigators observed better tumor control in the MC38 colorectal cancer with intravenous injection. This study showed that the vaccine administration route can affect the responding T cell states and that stem-like T cells generated better antitumoral effect compared to effector T cells in a therapeutic cancer vaccination. Interestingly, Sultan et al. [100] found out that intravenous route generated a higher T cell response compared to subcutaneous route. They adoptively transferred low numbers of naïve antigen-specific cytotoxic T lymphocytes and then immunized the mice intravenously, intramuscularly, or subcutaneously with peptide antigens and poly-ICLC, anti-CD40 adjuvants. Intravenous vaccination was more efficient at expanding antigen-specific cytotoxic T lymphocytes compared to intramuscular and subcutaneous vaccination. The intravenous route can also be used to target tumors for in situ vaccination. Zhang et al. [111] developed a nanoparticle platform containing poly-[(N-2-hydroxyethyl)-aspartamide]-Pt(IV)/β-cyclodextrin and CpG that accumulate in the tumor after intravenous administration and dissociate to release poly-[(N-2-hydroxyethyl)-aspartamide]-Pt(IV)/β-cyclodextrin for tumor killing. The resulting tumor antigens and CpG activate DCs and prime T cells.

Other less common routes of peptide-based cancer vaccine delivery include oral delivery [112] and intratumoral delivery [113]. The choice of administration route should be carefully considered to balance between effectiveness and potential side effects.

## Evaluation of Cancer Vaccines

### Preclinical tumor models

Most tumor models in preclinical cancer vaccine development are implanted subcutaneously for ease of monitoring tumor growth. However, subcutaneous models may not recapitulate the tumor microenvironment as well as orthotopic models. A recent study showed CMT 167, a lung carcinoma model, had fewer immune cells when implanted orthotopically compared to subcutaneously. However, no immune cell difference was observed in the 4T1 breast cancer model between orthotopic and subcutaneous tumors [114]. The commonly used preclinical tumor models for vaccine evaluation are summarized in Table 2.

**Table 2.** Common preclinical models for cancer vaccine development

Cell line	Tumor type	Mouse strain	Antigen	Sequence for MHCI epitope	Sequence for MHCII epitope
B16-OVA	Melanoma	C57BL/6	OVA	SIINFEKL [139]	ISQAVHAAHAEINEAGR
E.G7-OVA	Lymphoma	C57BL/6	OVA	SIINFEKL	ISQAVHAAHAEINEAGR
B16F10	Melanoma	C57BL/6	gp100, Trp2	Trp2: VYDFFVWL [116] gp100: EGSRNQDWL[140] B16-M27: REGVELCP GNKYEMRRHGTTHSLVIHD [117]	Trp1: CRPGWRGAACNQKI B16-M30: PSKPSFQE FVDWENVSPELNSTDQPFL
MC38	Colon carcinoma	C57BL/6	Adpgk, Reps1	Adpgk: ASMTNMELM Resp1: AQLANDVVL [118]	Ddr2: SEASEWEPHAVYFPLVLDDVNPS Pcdh18: SPWAYITTVTATDPDL Zmiz1: RPPADFTQPAASAAAAA [141]
TC1	Cervical carcinoma	C57BL/6	E7	E7: RAHYNIVTF [142]	E7: EQLNDSSEEEDTDEID [143]

Some tumor cell lines such as B16 and E.G7 are modified to express OVA, a commonly used model antigen in cancer research that is well-characterized with defined epitopes. In addition, many in vitro and in vivo assays have been developed for OVA-derived antigens. However, OVA is a foreign antigen that is not naturally present in mice and humans, and therefore is less clinically relevant. Two neoantigens that have been validated for the B16F10 melanoma model are gp100 and Trp2 [115,116]. The Sahin group used NGS to identify mutations in the B16F10, CT26 and 4T1 tumors and demonstrated tumor control with immunization of some synthetic 27-mer peptides, including B16-M30 [117]. For the MC38 tumor model, Adpgk and Reps1 are commonly used neoantigens. Yadav et al. [118] discovered mutations in the neo-epitopes within Adpgk and Resp1 proteins and demonstrated that immunization with the mutated peptides slowed MC38 tumor growth.

Among the different preclinical tumor models, TC-1 cervical carcinoma is relatively easy to treat; several vaccines generated long-term survival in mice bearing TC-1 tumors [9,45]. However, B16F10 tumors are highly aggressive and immunosuppressive and can metastasize to distant sites. While a few studies showed initial control of tumor growth after vaccination, tumors continued to grow after treatment cessation [55,119]. Poorly immunogenic tumors like B16 express lower levels of T cell costimulatory molecules and respond poorly to immunotherapies [120]. Cancer vaccines often need to be combined with immune checkpoint blockade such as anti-PD-1, anti-programmed death-ligand 1 or anti-cytotoxic T-lymphocyte-associated protein 4 antibodies in these tumor models [82,98].

The antitumor response can be examined in either a prophylactic model, whereby tumors are inoculated after vaccination, or a therapeutic model, whereby vaccination is given to treat established tumors. Most research focuses on therapeutic models for clinical relevance, although some formulations have been reported to be effective in both the prophylactic and therapeutic settings [101,119].

### DC cross-presentation and activation

In vitro DC cross-presentation assays using T cell hybridomas can be used as initial screening of vaccine candidates. B3Z cell is a T cell hybridoma that recognizes SIINFEKL, an OVA MHCI epitope, in the context of H-2K^b^ and produces β-galactosidase, which can be detected by adding fluorogenic or chromogenic substrates [121]. Similarly, BUSA14 is a T cell hybridoma that recognizes both human and mouse gp100 [25–33] peptides and produces β-galactosidase, and can be used in evaluating vaccines with gp100 neoantigen [122]. In vivo DC cross-presentation is evaluated by analyzing the amount of antigen-specific CD8^+^ T cells generated after vaccinations and will be discussed in the next section.

DC activation is another important criterion for vaccine efficiency. DCs up-regulate the expression of costimulatory molecules, such as CD80, CD86, CD83, and MHCII upon activation [123,124]. CD80 and CD86 are the most commonly evaluated DC activation markers that belong to the B7 family and bind to CD28 on T cells to enhance T cell activation by promoting T cell survival, expansion, and differentiation [125]. CD40 is another DC activation marker that binds to CD40L (CD154) on T cells and contributes to Th1 differentiation in CD4^+^ T cells [126].

### Antigen-specific T cells and T cell subsets

Cancer vaccines stimulate the adaptive immune system to generate T cells that can recognize cancer antigens. Therefore, the magnitude of antigen-specific T cells is an important indicator for cancer vaccine efficiency. The MHC multimer assay (usually tetramers or pentamers), with multiple MHC molecules held together with specific peptides to increase the avidity between the MHC/peptide complexes and TCRs, is often used to evaluate the frequency of antigen-specific T cells. Enzyme-linked immunosorbent spot and intracellular cytokine staining are often used together with the MHC multimer assay to evaluate the functionality of antigen-specific T cells. After isolation from immunized mice, T cells are cultured in vitro and stimulated with antigens to evaluate antigen-specific cytokine-producing T cells.

We investigated 20 recently published peptide-based vaccine papers but did not observe an obvious correlation between the percentage of antigen-specific T cells and antitumor capacity [9,42,43,45,51,52,54,55,58,61,63,69,82,95,98,99,119,127–129]. A number of different factors could contribute to this. First, the percentage of antigen-specific CD8^+^ T cells over total CD8^+^ T cells was reported from various sources, including blood, spleen, lung and LN. The antigen-specific T cell frequency is usually higher in blood samples/PBMCs than in spleens [129,130]. T cells in the blood are circulating and thus have a higher chance to encounter antigens presented by the APCs and in the peripheral tissues. However, within the same study, the percentage of antigen-specific T cells does correlate with outcome [43,51]. In a study with albumin-binding vaccines, Zhu et al. showed that the albumin/vaccine nanocomplexes containing CpG and Adpgk neoantigen showed a 14.1- and 13.6-fold higher frequency of antigen-specific CD8^+^ T cells compared to CpG + Adpgk and IFA(CpG + Adpgk) controls, and only albumin/vaccine nanocomplexes generated long-term survival in a MC38 tumor model, with 2 out of 16 mice surviving more than 30 d. The number of immunizations, treatment schedules, and time of T cell evaluation also affect the magnitude of T cell responses. The frequency of antigen-specific CD8^+^ T cells increases as more immunizations are given [98,99]. For instance, Kuai et al. demonstrated that the frequency of antigen-specific CD8^+^ T cells in PBMCs increased from 3% to 30% 1 week after the first immunization compared to 1 week after the third immunization [98]. The number gradually decreased after the treatment stopped [69,98]. Therefore, optimizing the dosing schedule could generate a better immune response. We suggest that the field could standardize one of the time points of evaluation, such as 7 d after the second immunization, for better comparison between formulations from different research groups.

T cell states and subsets also affect therapeutic efficacy. Baharom et al. [101] used different injection routes to generate similar magnitude of antigen-specific T cell responses but demonstrated better therapeutic efficacy when T cells were more stem-like. Similarly, Ramirez-Valdez et al. [130] also found that the extent of CD8^+^ T cell response was not the only factor determining tumor control. They either primed the mice with nanoparticles containing irrelevant antigen followed by boosting with adenoviral vector containing cancer antigen, or primed the mice with nanoparticles containing cancer antigen followed by adenoviral vector containing irrelevant antigen. They observed that the 2 methods generated similar magnitudes of antigen-specific CD8^+^ T cell response 1 week post the vaccine boost but differed significantly in a therapeutic MC38 tumor model, suggesting that the innate immunity could play a role. CD8^+^ T cells have different subsets, and only Tc1, Tc2, and Tc22 have cytotoxic function [131]. Regulatory T (Treg) cells, a subset of CD4^+^ T cells, hinder antitumor responses by secreting inhibitory cytokines, suppressing cytolysis, and impeding DC maturation [132]. To account for the immunosuppressive T cells, some people used the CD8^+^ T cells to the Tregs ratio for analysis [55]. Therefore, it is important to examine both the magnitude of T cell response and T cell states to predict the therapeutic outcome.

## Conclusion and Perspectives

Peptide-based cancer vaccines hold great promise as a novel cancer immunotherapy. Peptides are safe, easy to make, and cost-effective and have good storage stability. With the development in bioinformatics and machine learning, neoantigens can be selected and personalized for different patients for optimal care. Development of new delivery platforms enhances antigen delivery and presentation, resulting in improved antigen-specific T cell responses and antitumoral efficiency. Discovery of new adjuvants boosts the immune response and further enhances the activity of the cancer vaccines.

Despite recent progress in cancer vaccine development, most formulations are still in the preclinical stage. Many cancer vaccine studies use the model antigen OVA to test the delivery platforms, but its translational value is limited. To bring cancer vaccines into the clinical stage, more research needs to be done to identify antigens that can effectively bind to the MHC molecules and are specific to tumors. In addition, cancer vaccines have limited efficacy in some preclinical models, especially in nonimmunogenic and aggressive tumor models. While many vaccines slow tumor growth, they may not lead to tumor elimination. Cancer vaccines may need to be combined with traditional cancer treatment methods, such as chemotherapy or radiation therapy to induce cancer cell death and release antigens to further boost the immune response. In addition, cancer vaccines may be combined with immune checkpoint inhibitors such as anti-PD-1 and anti-cytotoxic T-lymphocyte-associated protein 4 to enhance T cells’ capacity to fight against cancer cells. Because T cell exclusion in the tumor microenvironment might also contribute to vaccine failures, approaches for promoting T cell infiltration into tumors is paramount. Many cancer vaccine studies focus on the quantity of antigen-specific T cells, but investigation into the quality of the T cells will provide more insights for future vaccine development. T cells are not the only players in tumor immunity; the tumor microenvironment contains other immune cells such as myeloid-derived suppressor cells, Treg cells and tumor-associated macrophages that are immunosuppressive. It is critical to eliminate or reprogram the immune-suppressive cells to turn a “cold” tumor into a “hot” tumor to optimize the activity of antigen-specific T cells. Furthermore, optimizing vaccine adjuvant dosing, timing, and combinations can yield additional benefits. Although studies have shown the benefits of combining multiple adjuvants, it is not clear what combination yields the best results and what the best timing is for activating the innate immune pathways. Achieving these goals will be important for developing more effective cancer vaccines and translating them into clinical therapies.
